# How a supply‐side intervention can help to increase caesarean section rates in Burkina Faso facilities—Evidence from an interrupted time‐series analysis using routine health data

**DOI:** 10.1111/tmi.13840

**Published:** 2022-12-23

**Authors:** Marion Ravit, Julia Lohmann, Alexandre Dumont, Charles Kabore, Jean‐Louis Koulidiati, Manuela De Allegri

**Affiliations:** ^1^ Emergency Obstetric and Quality of Care Unit Liverpool School of Tropical Medicine Liverpool UK; ^2^ Université Paris Cité IRD, Inserm, Ceped F‐75006 Paris France; ^3^ London School of Hygiene & Tropical Medicine UK; ^4^ Heidelberg Institute of Global Health University Hospital and Medical Faculty, Heidelberg University Germany; ^5^ Institut de Recherche en Sciences de La Santé Ouagadougou Burkina Faso; ^6^ Institut supérieur des sciences de la santé Université Nazi Boni Bobo‐Dioulasso Burkina Faso

**Keywords:** Burkina Faso, caesarean section, health policy, low‐income countries, maternal health, performance‐based financing, user fees

## Abstract

**Objectives:**

In Burkina Faso, only 2.1% of women give birth by caesarean section (CS). To improve the use of maternal health services during pregnancy and childbirth, many interventions were implemented during the 2010s including performance‐based financing (PBF) and a free maternal health care policy (the gratuité). The objective of this study is to evaluate the impact of a supply‐side intervention (PBF) combined with a demand‐side intervention (gratuité) on institutional CS rates in Burkina Faso.

**Methods:**

We used routine health data from all the public health facilities in 21 districts (10 that implemented PBF and 11 that did not) from January 2013 to September 2017. We analysed CS rates as the proportion of CS performed out of all facility‐based deliveries (FBD) that occurred in the district. We performed an interrupted time series (ITS) analysis to evaluate the impact of PBF alone and then in conjunction with the gratuité on institutional CS rates.

**Results:**

CS rates in Burkina Faso increased slightly between January 2013 and September 2017 in all districts. After the introduction of PBF, the increase of CS rates was higher in intervention than in non‐intervention districts. However, after the introduction of the gratuité, CS rates decreased in all districts, independently of the PBF intervention.

**Conclusion:**

In 2017, despite high FBD rates in Burkina Faso as well as the PBF intervention and the gratuité, less than 3% of women who gave birth in a health facility did so by CS. Our study shows that the positive PBF effects were not sustained in a context of user fee exemption.

## INTRODUCTION

The number of maternal deaths has considerably decreased in the world since the 1990s, from 390,155 in 1990 to 275,288 in 2015. Almost half of maternal deaths occur in sub‐Saharan Africa (SSA), where the maternal mortality ratio (MMR) remains substantially higher (374.9 per 100,000) than the global average (195.7 per 100,000) [1]. Most of the complications during pregnancy and childbirth leading to women's deaths are preventable or treatable [2]. At delivery, severe complications may require a caesarean section (CS), an intervention that has been recognised as effective in saving the lives of mothers and children if practiced when medically justified (WHO 2015) [3]. Although optimal CS rates are heavily debated, we know that a population CS rate of less than 5% does not cover all obstetrical needs [3]. However, a study conducted in West Africa concluded that in 2015 only 3% of women delivered by CS [4].

The determinants of the use of maternal health services, and of CS in particular, are multiple. On the one hand, they relate to demand for care, whereby individual, household, and contextual factors influence the decision to seek care and the identification of and access to health facilities. Among these factors, affordability has been unanimously identified as playing a major role in shaping access to maternal health care in SSA [5, 6, 7]. On the other hand, they relate to the supply of health services, since what care is available and functional determines whether a woman receives appropriate treatment to meet her health needs. Among supply‐side determinants of access, the availability and functionality of health services and lack of human resources are among the most commonly cited barriers to accessing appropriate care, including CS [8].

In Burkina Faso, to improve women's access to care during pregnancy and childbirth, a national user fee reduction policy for delivery (80% subsidy) was introduced in October 2006 for CS, and in April 2007 was expanded to cover all deliveries, with and without complications [9]. Evidence indicates that the introduction of this policy has resulted in substantial increases in the rate of facility‐based delivery (FBD) [10, 11, 12]. In June 2016, the national user fee reduction policy (80% subsidy) for all deliveries was extended to a free maternal health care policy. The gratuité covers all care for pregnant women, childbirth and CSs if needed [13]. Many studies in SSA have shown a positive impact of free maternal health care on FBDs in health facilities [11, 14, 15]. However, while the rate of FBD has reached 80% in Burkina Faso, the rate of CS among delivering women remains low, far from the minimum of 5% recommended by WHO, at an estimated 2.1% [16, 17].

Alongside user fee reduction and removal policies, performance‐based financing (PBF) was introduced in Burkina Faso in the 2010s. The objective was to improve access and quality of health services by financially incentivising health providers based upon their performance. PBF has been piloted in a large number of countries particularly in SSA over the last years, but a recent Cochrane review concludes that accompanying impact evaluations remain uncertain about PBF impact on the utilisation of institutional and CS deliveries [18]. In Burkina Faso, a recent population‐based study showed that PBF had a modest positive impact on the utilisation of FBD [19]. Due to sample size restrictions, the study was unable to analyse CS specifically.

The question we are asking here is whether the combination of these two interventions had an effect on CS rates and what that effect was. The positive results for institutional delivery, combined with those due to the removal of financial barriers under the gratuité, lead us to assume that more women benefited from CS. We assumed that as more deliveries take place in health facilities, more women would be referred to a health facility for CS if needed. The present study offers an opportunity to focus on CS specifically by using routine health service provision data from the health management information system. We assessed whether a supply‐side intervention (PBF) combined with a demand‐side intervention (the gratuité) led to an increase in the rate of CS in Burkina Faso, using a quasi‐experimental approach.

## MATERIAL AND METHOD

### Context

Burkina Faso is a landlocked, low‐income country in SSA. Its population was estimated at 18.6 million in 2016 [20]. According to the Human Capital Index, Burkina Faso is among the least developed countries in the world, ranking 144th out of 157 countries [20]. Even if maternal mortality has decreased in Burkina Faso the past 20 years, the MMR remained very high in 2015 (352 maternal deaths per 100,000 live births) [1].

The Burkinabé public health care system is pyramidal [13]. Basic Emergency Obstetric Care Centres (BEmOC) provided by Centres médicaux (CM), Centres de Santé et de Promotion Sociale (CSPS) and dispensaries constitute the first level of care in rural areas.

CS are provided only at Centres médicaux avec Antenne chirurgicale (CMA, or district hospitals), Centres Hospitaliers Régionaux (CHR, or regional hospitals) or Centres Hospitaliers Universitaires (CHU, university hospitals), which provide Comprehensive Emergency Obstetric Care Services (CEmOC). By 2015, 47 CMAs, 9 HRs and 4 CHUs were available in the country [13].

### PBF intervention

From 2014 to 2018, the PBF programme was rolled out in six regions of Burkina Faso (Centre Nord, Centre Ouest, Nord, Sud Ouest, Boucle du Mouhoun, and Centre Est). Within each region, two districts were selected to receive PBF based on their particularly poor outcomes. The PBF intervention also focused on equity by testing whether PBF in combination with demand‐side interventions may additionally improve pro‐poor access to health care services [21]. As the primary objective of the PBF programme was to improve the utilisation and quality of maternal, newborn and child health services (MNCH), the PBF benefit package covered a broad range of MNCH services. Facilities received case‐based incentive payments, including between 1000 and 4800 Franc Communauté Financière Africaine (FCFA) for each vaginal delivery, and 10,000 and 15,000 FCFA for each CS performed, with variations in incentive amounts related to the level of care and other facility‐ and patient‐related considerations. Quality was assessed with comprehensive quality checklists and facilities received an additional quality bonus if they achieved a quality score above 60% [22].

### Study design

We performed an Interrupted Time Series Analysis analysis to evaluate the impact of PBF alone and then in conjunction with the gratuité on CS rates using the method developed by Linden [23].

The ITS design is often used for the evaluation of public health interventions. The principle is to make a pre‐post comparison accounting for underlying trends in the outcome [24], using data collected at a regular interval over time. In this study, the objective was to evaluate if the change in the level and/ or trend in the outcome variable (here the CS rate) is the same for both the facilities that did not implement PBF (the control group) and the facilities that did implement PBF (the intervention group). As the gratuité was implemented indistinctly in all facilities, we evaluated whether there was a change in the level and/or trend of the CS rate after the introduction of this policy. We also assessed whether the impact was different depending on the implementation of PBF in the hospitals and therefore whether the combination of the two interventions had an impact on CS.

### Data and their sources

We used monthly facility‐level data from the routine health management information system (HMIS) in the 24 districts where the PBF programme was rolled out, from January 2013 to September 2017. We excluded three districts with more than 80% of missing data (facility‐month) on the first year (our pre‐intervention period). Data from 21 districts were therefore included in the study: 10 districts where the PBF programme was implemented and 11 districts where it was not.

### Outcomes

To capture the change not only at the population level, our outcome variable was defined as the proportion of CS of all FBD having taken place in a district. For each month in each district, the CS rate corresponds to the total number of CS performed in the district's CMAs or CHRs divided by the number of deliveries that were reported for all facilities in this district (including CSPS, CMA and CHR). Data were aggregated for all facilities within the intervention group and the control group.

### Exposure

We compared trends in CS rates between the 10 PBF districts and the 11 non‐PBF districts. We studied for each group 12‐time points (months) before the introduction of the PBF intervention (January 2013 to December 2013), 30 after the introduction of the PBF and before the introduction of the gratuité (January 2014 to May 2016) and 15 time points after the introduction of the gratuité, when both interventions were present (June 2016 to September 2017).

### Statistical analysis

We performed ITS analysis to evaluate if the PBF alone and then in conjunction with the gratuité had an impact on CS rates using the method developed by Linden [23]. In this study, the regression model equation for the two‐group analysis is the following:

$$Y_{t}=\beta_{0}+\beta_{1}T_{t}+\beta_{2}\;{\mathrm{PBF}}_{t}+\beta_{3}T_{t}{\mathrm{PBF}}_{t}+\beta_{4}\;Z+\beta_{5}\;ZT_{t}+\beta_{6}\;Z{\mathrm{PBF}}_{t}+\beta_{7}\;ZT_{t}{\mathrm{PBF}}_{t}+\beta_{8}\;\text{gratuité}+\beta_{9}\;\text{gratuité}X_{t}+\beta_{10}\;{\text{gatuité}}_{t}\;X_{t}+\varepsilon_{t}$$

$Y_{t}$ = CS rates.

$T_{t}$ = time since the start of the study.

${\mathrm{PBF}}_{t}$ = dummy variable representing the PBF intervention period (preintervention = 1, otherwise = 0).

${\text{gratuité}}_{t}$ = dummy variable representing the PBF + gratuité intervention (preintervention = 1, otherwise = 0).

Z = a dummy variable to denote the group: treatment group (PBF) vs control group (no PBF).

$\beta_{0}$ = the intercept (starting level of the outcome variable).

$\beta_{1}$ = the slope (trajectory of the outcome variable until the beginning of PBF).

$\beta_{2}$ = the change in the level of the outcome that occurs in the period immediately after the introduction of PBF.

$\beta_{3}$ = the difference between pre‐ and post‐intervention slopes.

$\beta_{4}$ = difference in the level between treatment and control prior to PBF intervention.

$\beta_{5}$ = difference in the slope between treatment and control prior to PBF intervention.

$\beta_{6}$ = difference between treatment and control in the level of the outcome immediately after PBF.

$\beta_{7}$ = difference between treatment and control in the slope after initiation of the PBF intervention compared with preintervention (difference‐in‐differences of slopes).

$\beta_{8}$ = difference in the slope between treatment and control prior to gratuité intervention.

$\beta_{9}$ = difference between treatment and control in the level of the outcome immediately after the gratuité.

$\beta_{10}$ = difference between treatment and control in the slope after initiation of the gratuité compared with preintervention (difference‐in‐differences of slopes).

We conducted the ITS analysis using an ordinary least squares regression model with Newey‐West standard errors to address autocorrelation and possible heteroscedasticity. The Cumby‐Huizinga test was used to check whether the model estimates took into account the correct autocorrelation structure. Model estimates were also plotted for the visual inspection of actual levels and expected trends and levels of CS rates on three periods: before the introduction of any intervention, after the introduction of PBF and after the introduction of the gratuité.

Although we excluded three CMAs due to a large proportion of missing values, our remaining sample still included a small number of missing values (less than 3% for deliveries and 10% for CS), which were handled using multiple imputation. We followed a two‐step procedure. First, we imputed mean values for deliveries and then used data including these imputed mean values as our independent variable to impute CS. Five rounds of imputations were done in Stata using the mi impute Poisson command [25] and the resulting average values were then used for analysis. Analyses were performed using Stata version 13.0 software (Stata Corp., College Station, TX, USA).

### Ethics statement

This study presents no major ethical challenges since it is based on an analysis of fully anonymous secondary data. The study received ethical approval from the Ethics Commission of the Medical Faculty of the University of Heidelberg (approval number S‐272/2013) and the Ethics Committee of the Burkina Faso Ministry of Health (approval number 2017‐9‐138). The Burkina Faso Ministry of Health granted written permission to download and use the data for the analysis.

## RESULTS

### Descriptive analysis

**FIGURE 1:**
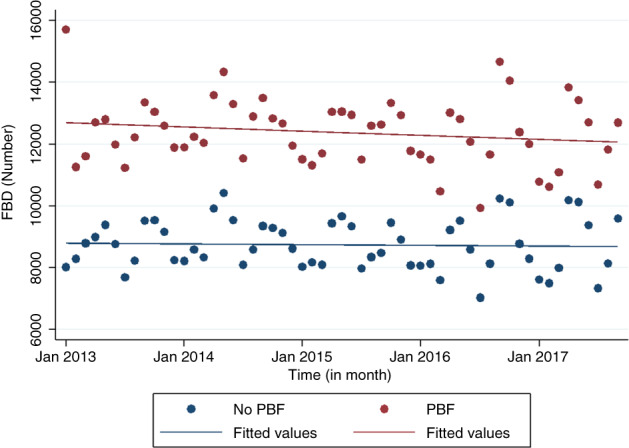
Evolution of the CS rates in 21 districts (10 that experimented PBF, 11 that did not) by month from January 2013 to September 2017 (observations and fitted lines)

**FIGURE 2:**
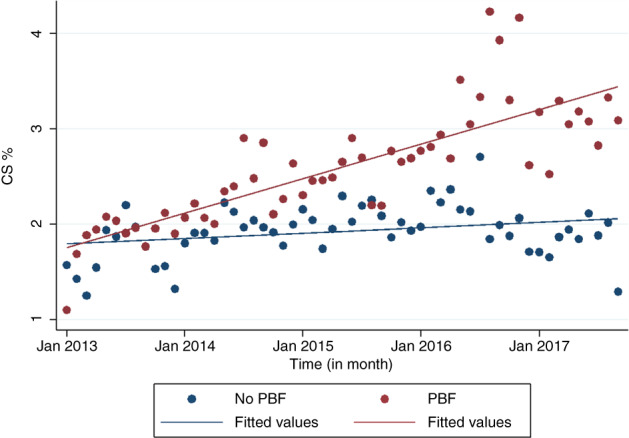
Evolution of the CS rates in 21 districts (10 that experimented PBF, 11 that did not) by month from January 2013 to September 2017 (observations and fitted lines)

Figure 1 shows the monthly evolution of the number of FBDs in the 21 districts (10 that experimented with PBF, 11 that did not) throughout the study period, revealing that the trend in the number of FBDs tends to be relatively stable over time in both groups. Figure 2 shows a very slight increase in the CS rate over time in both groups of districts, those which experimented PBF and those which did not.

Table 1 presents a description of the sample. The HMIS data available for the 21 districts over the 47‐month period (from January 2013 to September 2017) allowed us to analyse 1,203,296 deliveries, including 27,846 CS. Before the introduction of the PBF intervention, the rate of CS performed at CMA and CHR facilities among deliveries in the district was 1.77%, with a slightly higher rate among facilities that received the PBF intervention (1.85%) than among those that did not receive it (1.66%). CS rates gradually increased over time, reaching 2.70% after the introduction of gratuité.

**TABLE 1:** Descriptive statistics (from January 2013 to September 2017)

	Number of districts	Number of deliveries	Number of CS	CS (%)	Before PBF	After PBF	After gratuité
					CS (%)	CS (%)	CS (%)
Total	21	1,203,296	27,846	2.31	1.77	2.33	2.70
PBF	10	705,309	18,271	2.59	1.85	2.53	3.27
No PBF	11	497,987	9575	1.92	1.66	2.04	1.91

### ITS analysis

**FIGURE 3:**
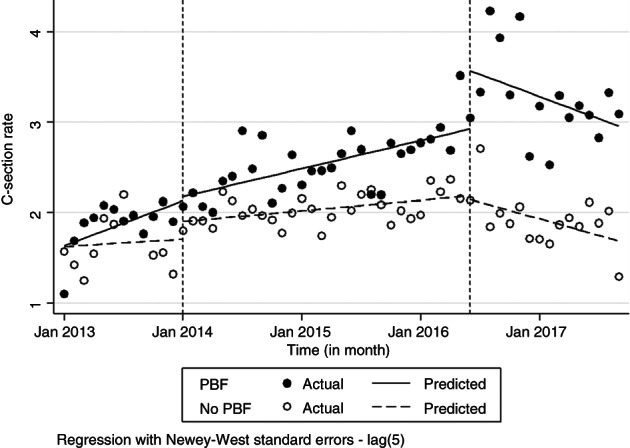
Level and slope changes in monthly CS rate by group (PBF and no‐PBF)

Table 2 presents the ITS regression model coefficients and 95% confidence intervals (CIs) for CS rates. Figure 3 shows the corresponding graphical visualisations of level and slope changes. The Cumby‐Huizinga test we performed showed that the regression model present autocorrelation at lags 4 and 5. We specified our model to account for this autocorrelation accordingly.

**TABLE 2:** Two‐group (PBF and No‐PBF) interrupted time series regression models for aggregated CS rates over 21 facilities

	Estimated CS rates [95%CI]
Pre‐intervention period	
Intercept (no PBF group)	1.63 [1.30; 1.96]***
Initial difference between PBF and no PBF facilities	0.01 [−0.44; 0.46]
Trend (no PBF group)	0.01 [−0.05; 0.06]
Difference in the trend between PBF and no PBF facilities	0.04 [−0.03; 0.10]
Introduction of PBF intervention	
Change in the level (no PBF group)	0.19 [−0.21; 0.60]
Difference in the level between PBF and no PBF facilities	−0.15 [−0.64; 0.34]
Monthly trend change (no PBF group)	0.00 [−0.05; 0.06]
Difference in the trend change between PBF and no PBF facilities	−0.02 [−0.09; 0.05]
Post‐intervention trend	
PBF group	0.03 [0.01; 0.04] ***
No PBF group	0.01 [0.00; 0.02]**
Trend difference	0.02 [0.00; 0.03]**
Introduction of gratuité (PBF + gratuité)	
Change in the level (no PBF group)	−0.04 [−0.25; 0.17]
Difference in the level between PBF and no PBF facilities	0.68 [0.20; 1.17]**
Monthly trend change (no PBF group)	−0.04 [−0.07; −0.01]**
Difference in the trend change between PBF and no PBF facilities	−0.03 [−0.07; 0.02]
Post‐intervention trend	
PBF group	−0.04[−0.07; −0.01]**
No PBF group	−0.03 [−0.05; −0.01]**
Trend difference	−0.01 [−0.05; 0.03]

Abbreviation: PBF, performance‐based financing.

*** *p* < 0.001.

** *p* < 0.005.

The CS rates at baseline (in January 2013) were similar between the two groups, estimated at 1.63% for the non PBF group and 1.64% for the PBF group. The difference in the mean baseline slope between the two groups was not significant meaning that they are comparable on the baseline trend. There is no statistically significant effect either after the first month of the PBF intervention or in the trend change over the period after the introduction of the PBF compared with before. However, the post‐trend analysis shows that the CS rates increased monthly by 0.03% points after the introduction of PBF in the PBF group and by 0.01% points in the non PBF group. The difference between the two groups was significant meaning that CS rates increased significantly more in the PBF group after the introduction of PBF intervention.

A significant difference was observed between the two groups immediately after the introduction of the gratuité alongside PBF as well as in the trend change. After the introduction of the gratuité, CS rates decreased significantly each month by 0.04% points in the group of facilities where the PBF was introduced and by 0.03% points in the non PBF group, with no significant differences between the two groups.

## DISCUSSION

To our knowledge, this study is the first to examine the impact of a supply‐side intervention (PBF) combined with a demand‐side intervention (gratuité) on institutional CS rates in Burkina Faso. We found that CS rates increased more in districts that experienced PBF than in those that did not after the introduction of this intervention. However, after the introduction of the gratuité, CS rates decreased in all districts, independently of the PBF intervention.

Our analysis shows that CS rates in Burkina Faso were very low at baseline in 2013. Less than 2% of women that gave birth in a facility had experienced a CS. The level and trend were not different between the two groups (PBF and no PBF), even though we can observe a slight increase of CS rates in the PBF group prior to the intervention. After the introduction of the PBF intervention, no immediate change in the level was observed, and the trend change was not different from before. However, we observed that during the period after the introduction of PBF, CS rates increased monthly by 0.02% points more in the PBF group than in the non PBF group.

In the absence of data on the mechanisms of change, we assume that the impact of PBF on CS rates was driven by a combination of enhanced referral capacity and motivation at primary‐level facilities and enhanced capacity at secondary‐level facilities. These emerging hypotheses would need to be verified in further research, possibly through qualitative inquiry.

However, even with PBF, CS rates fell far short of the 5% benchmark stipulated by the WHO. Again, in the absence of additional data, we can only speculate on the reasons. First, the incentive structure built within PBF might have not been sufficiently powerful to effect a dramatic change in the referral and obstetric care delivery practice. PBF aims to improve the quality of care by improving availability of inputs and equipment. Material resources are one of the best‐known barriers to access to obstetric care. A literature review conducted in 2013 addresses the barriers that contribute to the delay in caring for pregnant women in health structures in developing countries [8]. Of the 43 studies included, 38 identify problems related to the availability of drugs, functional equipment, or blood for transfusion. These items are particularly important in the treatment of obstetric complications, especially in the case of a CS. In Burkina Faso, the lack of material resources to perform a CS is particularly problematic. For instance, in 2007, only 20.9% of district hospitals had blood banks, leaving patients to rely on volunteers or family members in case of need [26].

Second, CS as a surgical intervention requires substantial material and human resources, both of which tended to be lacking in Burkina Faso prior to the intervention. PBF aims to improve the quality of care by improving the availability of inputs and equipment. A literature review conducted in 2013 addresses the barriers that contribute to the delay in caring for pregnant women in health structures in developing countries [8]. Of the 43 studies included, 38 identify problems related to the availability of drugs, functional equipment, or blood for transfusion. These items are particularly important in the treatment of obstetric complications, especially in the case of a CS. A recent study showed that in Burkina Faso, a PBF programme had no impact on the availability of essential medicine due to contextual constraints and other policies implemented in the country [27]. In Burkina Faso, the lack of material resources to perform CS is particularly problematic. For instance, in 2007, only 20.9% of district hospitals had blood banks, leaving patients to rely on volunteers or family members in case of need [26].

Although the cash inflow through PBF should have enabled facilities to improve at least their material resource situation slightly, human resource availability was largely beyond the scope of PBF and might have continued to constitute a major bottleneck in higher‐order service provision, including CS. Through incentives, PBF aimed at improving health care providers' motivation. It is essential to improve the capacity of health care providers to assess the condition of women in labour and to improve their decision‐making about whether to perform CS. Burkina Faso suffers from a severe lack of qualified human resources such as gynaecologists and surgeons. About half of the district hospitals, such as CMAs, did not even have two physicians with surgical skills, which means that it is effectively not possible to perform CS continuously. Furthermore, a higher availability of physicians and midwives was found in regional hospitals and in urban facilities than in district hospitals and in rural areas [26]. Midwives help women give birth and only ‘surgical attachés’, a nursing cadre with training in certain surgical methods including CS, are present to perform surgery in these structures. Although this is part of the official human resources strategy in Burkina Faso in light of the dire shortage of higher‐skilled cadres, their level of knowledge is clearly inferior to the level of knowledge of medical specialists such as gynaecologists and obstetricians, particularly in case of complications [28]. Beyond the capacity to perform CS at the CMA, the study by Comparé showed that in 80% of lower‐level facilities, no team member had been trained in the use of emergency obstetric care guidelines [26].

Third, as a reorientation of service provision towards higher CS rates not only concerns the supply side, but likely also touches on the cultural dimensions of childbirth, a more pronounced PBF effect might have simply needed more time to unfold.

After the introduction of the gratuité, we observed a change in trend and a decrease in CS independently of the PBF intervention. The supposed positive effect of the gratuité, which should encourage more women to attend a facility to give birth, was not followed by more CS. A recent study showed that in Burkina Faso, while a modest positive effect on health care utilisation was observed after the introduction of PBF, this effect was not accentuated after the introduction of free health care policy [29]. In the literature, only a few studies have looked at the impact of user fee exemption policies on CS in SSA. In contrast to us, most of them found a positive impact of these policies on CS rates in Mali, Benin and Senegal [11, 15, 30]. Only one study did not find any evidence of impact of this kind of policy on CS rates in Kenya, Ghana and Senegal [14]. The authors hypothesized that the geographical proximity of hospitals and the quality of available services may be more important determinants of the provision of CS than economic barriers such as user fees.

There are several limitations to this study. First, our sample is not representative of the whole country, as it only covered around one‐third of the country's districts, excluding the most remote districts as well as the major cities of Ouagadougou and Bobo‐Dioulasso. However, our study reflects the situation in the more rural areas of the country. Second, in this study, we do not have any information on the practice of CS or the conditions under which these procedures are performed. Previous studies estimated that between 12% and 24% of CS in Burkina Faso are non‐medically justified [31, 32]. A CS with no medical indication can have harmful consequences for the mother, especially in the context of developing countries [33, 34]. The data we used do not allow us to know if the small increase in institutional CS rates we found reflects an increase in medically justified CS, which would be a welcome development, or rather an increase in unnecessary and potentially harmful surgeries.

## CONCLUSION

In 2017, despite high FBD rates in Burkina Faso, the PBF intervention and the gratuité, less than 3% of women who gave birth in a health facility had a CS. This is far from the minimum of 5% recommended by the WHO. This study shows that after the implementation of a supply‐side intervention the capacity to perform a CS in low‐income settings can be improved. However, this is not sustainable in the context of gratuité, where more women are encouraged to deliver in health facilities. Both infrastructure and health care personnel need to be supported in a free policy context to ensure the capacity to respond to emergency obstetric care such as a CS.
